# Evaluating the potential toxicity of ampicillin using *Drosophila melanogaster* as a model organism

**DOI:** 10.1016/j.toxrep.2025.101992

**Published:** 2025-03-11

**Authors:** Asem Sanjit Singh, Dhruv Pathak, Sakshi Jain, Manoharmayum Shaya Devi, Upendra Nongthomba

**Affiliations:** a Developmental and Biomedical Genetics Laboratory, Department of Developmental Biology and Genetics, Indian Institute of Science, Bengaluru 560012, India; b ICAR-Central Inland Fisheries Research Institute, Barrackpore, West Bengal 700120, India

**Keywords:** *Drosophila*, Ampicillin, Antimicrobial Peptides, Epigenetics, Life-span, Methylation

## Abstract

Antibiotic resistance is an indispensable threat facing in the present era. However, the studies on long term and trans-generational effects of using drugs or antibiotics on living organisms are scarce. Emphasizing the necessity to address such problems, this study investigated the potential effects of antibiotic, ampicillin (AMP) stress on the physiology of *Drosophila melanogaster* across multiple generations with mechanistic details. We evaluated the larval feeding behavior, fertility, cell viability in ovary and testis, longevity, expression of methylation-related genes (*dDnmt2* and *dMBD2/3*), and antimicrobial peptide production. Larvae exposed to AMP exhibited increased mouth hook movement, indicating altered behaviour. AMP stress significantly reduced fertility across generations, with eclosion counts decreasing notably in F_3_ and F_4_ generations compared to controls. Moreover, AMP-treated flies showed decreased cell viability in ovary and testis, leading to impaired reproductive function. AMP exposure shortened the mean lifespan of flies and upregulated the expression of apoptosis-related gene *p53* in females. However, there was no significant difference in *p53* expression in males. Additionally, AMP stress caused a significant decrease in *Drosomycin* expression in treated males, while no significant changes were observed in *Drosocin* and *Metchnikowin*. In treated females, *Drosocin* and *Drosomycin* expression increased significantly, whereas the increase in *Metchnikowin* was not significant. The study also revealed downregulation of methylation-related genes (*dDnmt2* and *dMBD2/3*) in AMP-treated female flies which was normalised in the rescue flies suggesting disrupted epigenetic mechanisms. Overall, the findings highlighted the importance of evaluating the trans-generational impacts of AMP stress on *Drosophila* physiology and gene expression, particularly in reproductive function and epigenetic regulation. The study of the impact of widely used antibiotic, AMP on model organism, *Drosophila* (model organism known for its genetic similarity to human), will help in predicting potential impacts on higher organisms and human. The finding would ultimately promote proper use of antibiotics and use of alternative medicine.

## Highlights

• AMP stress reduces lifespan and fertility of *Drosophila melanogaster*, peaking in F_3_ and F_4_ generations in rescue flies.
• Low-dose AMP exposure alters epigenetic and apoptotic gene expression in a sex-specific manner.
• AMP effects persist across generations but normalize in rescue flies over time.
• Reproductive cell viability declines, with stronger effects in females.
• Immune response gene expression upregulated in female and downregulated in males under AMP stress.

## Graphical Abstract

**Figure:**
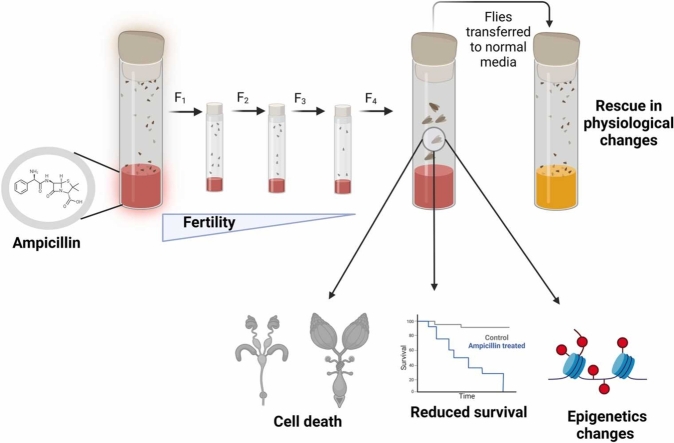

## 1 Introduction

Antibiotics are the third best-selling medicine globally. A significant portion of these, ranging from 25 % to 75 % of the doses administered, eventually enter the environment, contributing to pollution and predominantly being found in various ecosystems. The toxicity profile of antibiotics varies depending on compound composition, pathways, administration routes, and targeted genes/populations [1]. There is a surge in reports concerning antibiotic pollution highlighted persistent residues of antibiotics, including hydrophobic ampicillin, posing potential risks to human and animal hosts. The present study chose to assess the impacts of Ampicillin (AMP) on model organism, *Drosophila melanogaster*, as AMP is one of the most frequently used antibiotics [2], [3]. It is a micropollutant commonly found in food, water, sludge, fauna, and flora [4], [5]. They belong to the class of β-lactam antibiotics, exhibit broad-spectrum antimicrobial activity, and are prominently utilized as an intrapartum prophylactic against Group B *Staphylococcus* during pregnancy [6]. Its presence has been detected at concentrations as low as ng/L in effluents from municipal wastewater treatment plants and coastal waters [7], [8], while hospital effluents exhibit levels ranging from tens to hundreds of μg/L [9], [10]. Notably, urinary excretion following pharmaceutical consumption serves as a primary source of antibiotic introduction into sewage systems, with AMP concentrations ranging from 10–100 mg/L in urine [11]. The escalating risk associated with AMP stems from increased discharge into the environment, facilitating its bioaccumulation in humans and animals.

Pollutants like heavy metals [12], microplastics [13], antibiotics [14], induces epigenetic changes in living organisms. And, epigenetic alterations can persist across generations even after the removal of the initiating factor [15]. Studies have demonstrated that pollutants induce epigenetic changes in *Drosophila melanogaster* [16], [17]. DNA methylation, a pivotal epigenetic modification, apoptosis initiation, orchestrates specific gene silencing, and regulation of mitosis and transcription under stress such as cadmium, has been reported in *Drosophila melanogaster* [17]. The transgenerational changes in fly body weight attributing to epigenetic changes induced by low concentrations of bis (2-ethylhexyl) phthalate (DEHP) was observed by Chen et al. [18]. The effects of antibiotics on *Drosophila* are: high concentration of penicillin was found to reduce survivorship rates at various developmental stages [19], [20]; impairing the enzymatic antioxidant defense system, particularly in mitigating oxidative stress [21]; alteration of gut microbiota; negative impact on fertility and development; reduce in antimicrobial peptide production [22]. However, there is significant void on the long-term effects of antibiotic across multiple generations and the underlying epigenetic mechanisms associated with these interventions. On this backdrop, the present study aims at addressing the impact of low-dose antibiotic (AMP) pollution, which does not elicit observable phenotypic changes in the host but influences progeny across multiple generations in term of feeding behaviour of larvae, lifespan, fertility, cell viability, apoptosis, immunity, and DNA methylation. The findings would be an important source of information on probable impacts of AMP on higher animal and human. The study has significant implications in terms of promoting appropriate antibiotic use in health, animal husbandry or farming.

## 2 Material and methods

### 2.1 Culture of insect

The *Canton-S* strain of *D. melanogaster* flies was cultivated following the method of Singh et al. [22] with modifications. And, this method was followed for the successive generations. It comprised of, a conventional corn-based medium, comprising yeast, agar, sucrose, and corn flour was used within an incubator set at 25 ± 1℃ and 50–60 % humidity. The fly was grown in a 12:12-hour light/dark cycle and applied in successive generations.

### 2.2 Determination of concentration

The experimental dosage was determined based on the feeding behaviour demonstrated by larvae. It is the dose of antibiotic at which the feeding behaviour of the larvae is unaffected. Initially, ampicillin (CAS: 69–52–3; SRL) was dissolved in double-distilled water at a concentration of 10 mg/ml, then filtered using a syringe filter (0.2 micron). Afterwards, the solution was mixed into standard *Drosophila* food to achieve the specified concentrations (25, 50, 100, 150, and 200 µg/ml). The feeding behaviour of twenty-five 3rd instar *Drosophila* larvae was observed for durations of 10 seconds, for subsequent analysis. Control experiments comprised of *Drosophila* food prepared solely with double-distilled water.

### 2.3 Fertility measurements

**Fig. 1:**
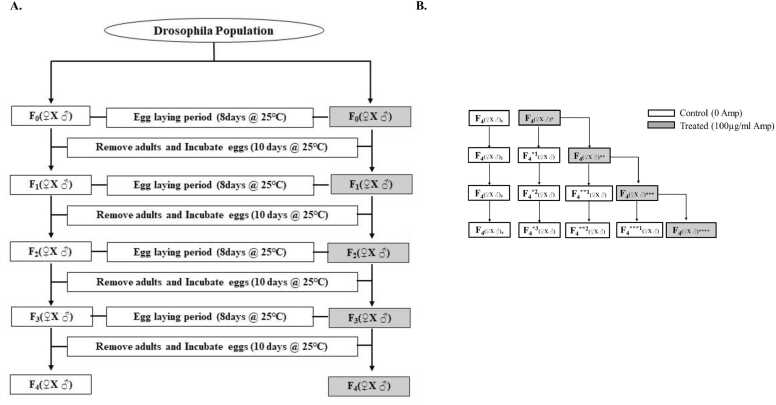
Schematic representation of the experimental setup for fertility measurement, longevity test, MTT assay, and analysis of *dDnmt2, dMBD2/3, p53, Drs, Dro* and *mtk* gene expression. **A.** Ampicillin treatment across generations in *Drosophila*. **B.** Ampicillin treatment and rescue experiment of the offspring of experiment A.

To assess the impact of ampicillin on *Drosophila* fertility across generations, seven-day-old treated and control adult flies were collected and crossbred under the following conditions: control males were paired with control females, while treated males were paired with treated females. The flies were allowed to lay eggs in corn medium vials for 8 days under strictly controlled conditions of 25 ± 1℃ and 60 %-70 % humidity. On the 8th day, the parental flies were removed, and the eclosion process was monitored for the following 10 days, spanning successive generations, as depicted in Fig. 1A.

For the rescue experiment, seven-day-old, fourth-generation treated flies were collected and crossbred as follows: treated males were paired with treated females and maintained in ampicillin-enriched media that produced F_4_* *_(♀x♂)_. In the rescue group, treated males were similarly paired with treated females but reared in a standard corn-based medium that produced F_4_*^1^_(♀x♂)_. In contrast, the control group comprised flies continuously propagated in conventional corn-based medium across successive generations (F_4(♀x♂)1_, F_4(♀x♂)2_, F_4(♀x♂)3_ and F_4(♀x♂)4_). These flies were also allowed to lay eggs in corn medium vials for 8 days under meticulously controlled conditions of 25 ± 1℃ and 60 %-70 % humidity. On the 8th day, the parental flies were removed, and the eclosion process was observed over the next 10 days for each vial, as shown in Fig. 1B. The rescue group (F_4_*^1^_(♀x♂)_) was reared in a standard corn-based medium for next two generation and it was represented as F_4_*^2^_(♀x♂)_ and F_4_*^3^_(♀x♂)_. F_4_* *_(♀x♂)_ generation was similarly subjected to rescue experiment as discussed above producing F_4_* *^1^_(♀x♂)_, F_4_* *^2^_(♀x♂)_ and F_4_* **_(♀x♂)_. The experiment ended by subjecting F_4_* **_(♀x♂)_ to rescue experiment producing F_4_* **^1^_(♀x♂)_ and F_4_* ** *_(♀x♂),_ as shown in Fig. 1B.

### 2.4 Longevity test

For the longevity study, 100 fruit flies of F_4_ generation were assessed as single sample, with three biological replicates in each group. The daily mortality of the flies was recorded. The mean lifespan represented the average survival duration of all flies in each group, while the median lifespan indicated the middle value of survival days for the group. The maximum lifespan was calculated as the average of the survival days of the longest living 10 % of the flies.

### 2.5 MTT(3-(4,5-demethylthiazole-2)-2,5-diphenyltetrazolium bromide) assay

The ovaries and testes of seven-day-old, F_4_ *Drosophila* female and male flies*,* cultivated under treatment conditions (100 µg/ml of AMP) and control, were dissected in ice cold phosphate-buffered saline. The tissues were treated with 0.05 % trypsin-EDTA (Thermo Fisher Scientific) to prepare single cell suspension. Cell viability was then assessed using a modified version of the method described by Rajak et al. [23], employing the MTT cell proliferation and cytotoxicity assay kit (Cat no. 33,611; SRL, India). A mixture of 90 µL of fresh culture solution and 10 µL of MTT solution was added before a 4-hour incubation period. The resulting formazan crystals were dissolved by adding 110 µL of dimethyl sulfoxide. Absorbance at 490 nm and optical density (OD) was measured using a microplate absorbance reader (Infinite M200 Pro Nano-quant, TECAN, Austria).

### 2.6 Expression analysis of dDnmt2, dMBD2/3, p53, Drs, Dro and mtk gene

Healthy adult male and female flies from the F_4_ generations were collected from both control and AMP-treated groups. Total RNA was isolated from the collected healthy fruit flies using the Trizole method [22], in three replicates per group. RNA integrity was confirmed through spectrophotometer reading and Agarose gel electrophoresis, and single-strand cDNA was synthesized using the Thermo first strand synthesis kit. The primer details of the respective target genes are provided in Table 1. *rp49* gene was used as an internal control and the target genes comprised of methylation *(dDnmt2, dMBD2/3*), apoptotic processes (*p53*), and antimicrobial peptides *(Drs, Dro, mtk*) [24]. Quantitative polymerase chain reaction (qPCR) was performed using the Real-Time PCR system (Quant studio 3) following the manufacturer’s instructions. The qPCR protocol included initial denaturation at 95℃ for 12 minutes, followed by 40 cycles of denaturation at 95℃ for 15 seconds, annealing at 60℃ for 20 seconds, and extension at 72℃ for 20 seconds, concluding with melt curve analysis. Cycle threshold (Ct) values were recorded and used to calculate fold changes as gene expression levels using the 2-ΔΔCt method, comparing treatment and control groups [24].

**Table 1:** Primers used for RT-qPCR amplification.

Target gene	Primer sequence (5’-3’)	Product size (bp)
*q-rp49*	F- AGATCGTGAAGAAGAAGCGCACCAAG R-CACAGGAACTTCTTGAATCCGG	206
*q-drosocin*	F-TTTGTCCACCACTCCAAGCAC R-ATGGCAGCTTGAGTCAGGTGA	188
*q-drosomycin*	F-ACCAAGCTCCGTGAGAACCTT R-TTGTATCTTCCGGACAGGCAG	129
*q-metchnikowin*	F-CGATTTTTCTGGCCCTGCT R-CCGGTCTTGGTTGGTTAGGAT	126
*dDnmt2*	F-AAAGGGACACGCAAGACAAG R-GCTCCAGCGATTCAATAAACT	150
*dMBD2/3*	F-CTCACTGCCCAAGACCATAC R-TAGCGGTTGTTCAGGATTCA	172
*p*53	F-CCCATCCAACCACTTAATTTGCG R-AAGGTGATTTTGACAGCGGAC	85

### 2.7 Statistical analysis

For gene expression analysis, fold change values were plotted and subjected to statistical analysis to discern significant differences between the treated and control groups using GraphPad PRISM 8. The multiple *t*-test was executed to compare the means between the treatment groups and the control group to check for significant disparities.

## 3 Results

### 3.1 Effects of ampicillin on larval feeding

**Fig. 2:**
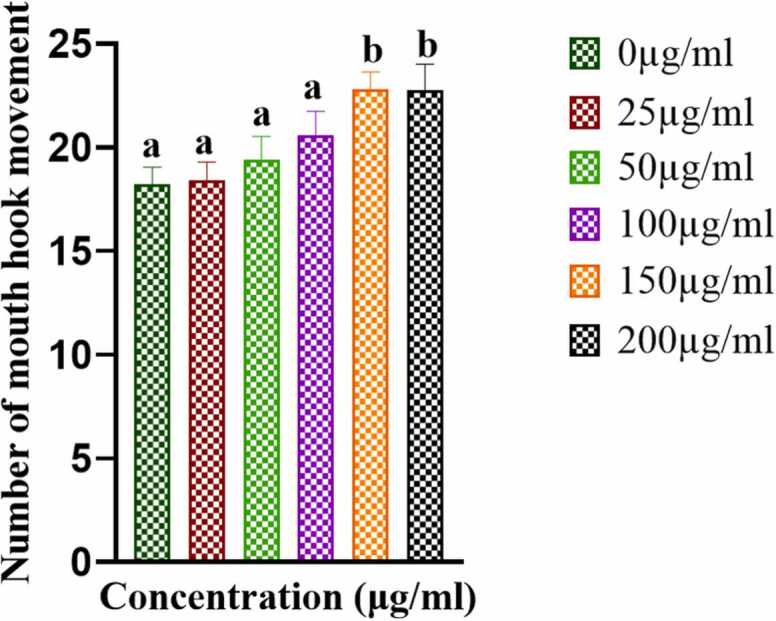
Mouth hook movement behaviour of third instar larvae. The bars with different letters indicate significant differences from each other; n = 100, p˂ 0.05.

A significant increase of 10-second in the mouth hook movement of third instar larvae was observed at the ampicillin concentration of 150 μg/ml (Fig. 2). There were no significant changes in feeding behaviour at 100 μg/ml and thus, 100 μg/ml was chosen as the maximum concentration for further experiments.

### 3.2 Effect of AMP on fertility

**Fig. 3:**
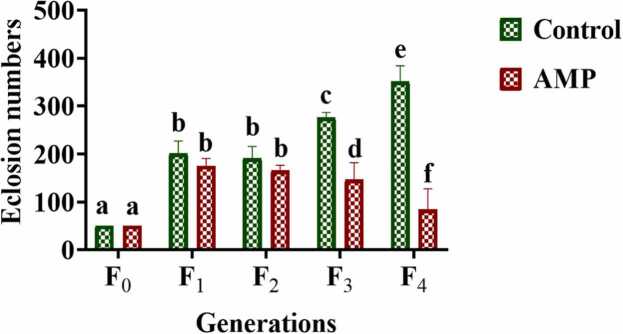
Fertility (in terms of eclosion number) of *Drosophila melanogaster* in treated and control groups over the generation. (F_0_ −0th generation; F_1_-1st generation, F_2_-2nd generation, F_3_-3rd Generation, F_4_-4th generation). The bars with different letters indicate significant differences from each other; n = 300, p˂ 0.05.

Following AMP treatment across each generation of flies, the eclosion count of their offspring decreased compared to that of the control group, indicating that AMP stress can impede *Drosophila* fertility. Post F_2_ generation of AMP treatment, the eclosion of the F_3_ generation decreased significantly compared to the control group (*p < 0.05*), signifying a decline in fertility due to AMP stress that could be inherited across generations. Upon continuous exposure to AMP in the F_4_ generation, fertility was notably reduced compared to the control group (*p < 0.01*) shown in Fig. 3. Then, the F_4_ generation flies were bring back to normal media to rescue, the decreased in fertility was passed down to the subsequent generation of rescue flies. Nevertheless, by the next two generation, fertility reverted to normal (F_4_*^3^_(♀x♂)_), as depicted in Fig. 8 (A).

### 3.3 Effects of AMP on the cell viability in ovary and testis

**Fig. 4:**
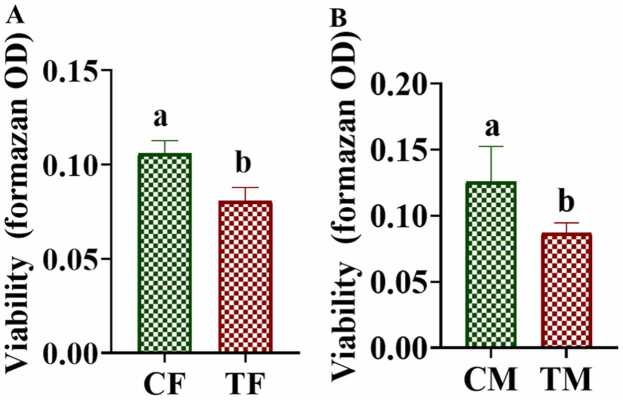
Change in the ovary cell viability (A) and testis cell viability (B) for 4th generation *Drosophila melanogaster* to Ampicillin (100 µg/ml). (CM-Control male; TM-AMP exposed male, CF-Control female; TF-AMP exposed female. The bars with different letters indicate significant differences from each other; p˂ 0.05.

The MTT assay was performed to understand the effects in the reproductive cells of the F_4_ generation flies. There was decreased in cell viability in ovary and testis following AMP treatment (Fig. 4). The OD of ovary in treated female was significantly lesser (*p ≤ 0.01*) than that of the control. Similarly, the OD of testis in treated male was significantly lesser (*p ≤ 0.05*) than that of control. The result shows that AMP treatment has a more pronounced detrimental effect on ovarian cells in females compared to the testis in males.

### 3.4 Effect of AMP on longevity

**Fig. 5:**
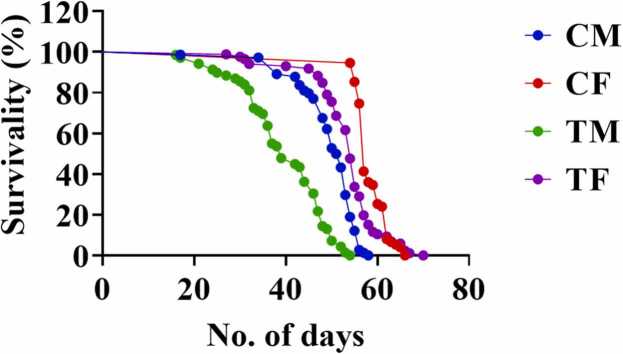
Longevity effects of Ampicillin. Abbreviations are as described in Fig. 4; n = 100, P ˂ 0.05.

The median lifespan of AMP-exposed flies was significantly reduced (*p < 0.0001*). Control and exposed female flies lived for 57 and 54 days respectively, while male flies had a median lifespan of 52 days (control) and 39 days (exposed), with significant differences (*p < 0.0001)* in both stages shown in Fig. 5. The maximum lifespan for control male, exposed male, control female, and exposed female flies was 56, 50, 62, and 61 days, respectively. Mean lifespans were 16, 15, 54, and 27 days, respectively. The results demonstrate that AMP exposure significantly shortens the lifespan of both male and female flies, with a more substantial impact on males. Exposed males live for 39 days, while exposed females live for 54 days. In contrast, control males and females live for 56 and 62 days, respectively. Control males and females begin to die at 16 and 54 days, respectively, whereas exposed flies begin to die at 15 and 27 days.

### 3.5 Effect of AMP stress on expression of dDnmt2 and dMBD2/3

**Fig. 6:**
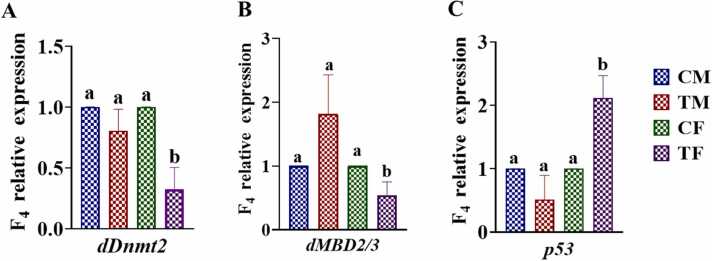
Relative expression of (A) *dDnmt2*; (B) *dMBD2/3* and (C) *p53* in F4 generation *Drosophila melanogaster*. Abbreviations are as described in Fig. 4. The bars with different letters indicate significant differences from each other; *p˂ 0.05*.

In F_4_ generation, the mRNA expression of methylation genes (*dDnmt2* and *dMBD2/3*) was significantly decreased (*p < 0.01*) in treated females, whereas, no significant difference was observed in AMP-treated male flies, as illustrated in Fig. 6. A and B respectively. The result shows that the AMP exposure, has more effect in the female flies with significant decreased in the *dDnmt2* and *dMBD2/3* expression while in male, they are not significant i.e. epigenetic changes are more prominent in the female flies*.* Then, the flies were removed from the antibiotic and bring back to normal media, the decrease in *dDnmt2 and dMBD2/3* was passed down to the subsequent generation (F_4_*^3^_(♀x♂)_ and F_4_* *^2^_(♀x♂)_) of rescue flies. Nevertheless, within the next two generations, methylation levels reverted to those observed in control flies, as illustrated in Fig. 8 (B: *dDnmt2* and C: *dMBD2/3B*).

### 3.6 Effect of AMP on p53

Following exposure to AMP stress, the expression of genes linked to apoptosis, specifically *p53,* was significantly upregulated (*p < 0.01*) in female flies, whereas in male flies, the upregulation was not significant when compared to the control group which means that the apoptotic activity was higher in the exposed female flies compared to male. The expression of *p53* in the F_4_ generation is depicted in Fig. 6 (C).

### 3.7 Effect of AMP on antimicrobial peptide production

**Fig. 7:**
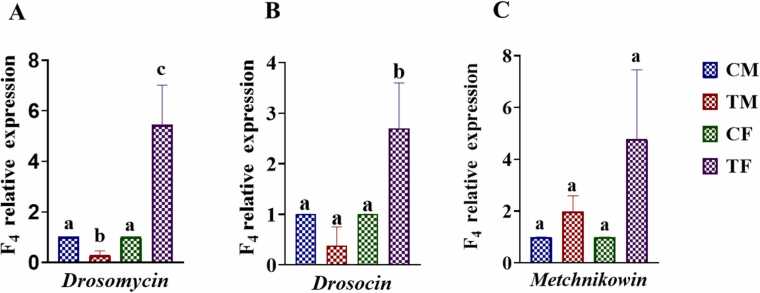
Relative expression of (A) *Drosomycin* (B) *Drosocin* and (C) *Metchnikowia* in F4 generation of *Drosophila melanogaster*. Abbreviations are as described in Fig. 4. The bars with different letters indicate significant differences from each other; *p˂ 0.05*.

Relative expression of *Drosomycin* and *Drosocin* revealed significant (*p < 0.05*) upregulation in the female flies while in male, significant (*p < 0.05*) downregulation was observed only in *Drosomycin.* No significant changes in *Metchnikowia*, levels were observed shown in Fig. 7. (A. *Drosomycin*, B. *Drosocin* and C. *Metchnikowia*). The result shows that AMP exposure significantly increased the production of *Drosomycin* and *Drosocin* in the female flies while it was decreased in the male flies.

**Fig. 8:**
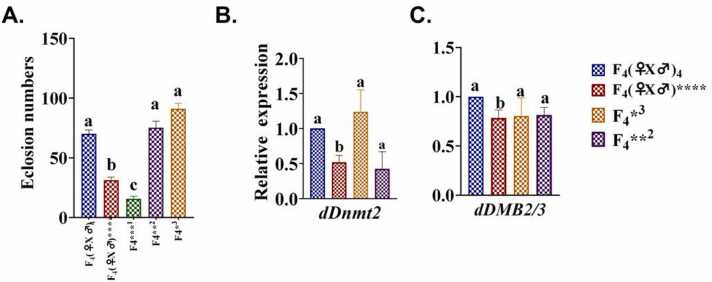
(A) Fertility of the F4 generation rescue flies (B) dDnmt2; (C) dMBD2/3 in F4 generation rescue *Drosophila* melanogaster. The bars with different letters indicate significant differences from each other; n = 100, p˂ 0.05.

## 4 Discussion

The outcomes of the current investigation revealed a notable reduction in the fertility and lifespan of *Drosophila* when exposed to AMP compared to control flies. This decrease could be attributed to the bioaccumulation of AMP during the exposure period. Remarkably, this finding resonated with previous studies on cadmium exposure [25], [26], which also reported the reduction in fertility and life span due to the shortening of telomeres in the treated group. It revealed an increase in the expression of telomeric repeat binding factor 1 (TERF1), suggesting that, it indirectly affected the length of telomeres by inhibiting the action of telomerase [27]. Sex, age, genetic background (epigenesis), and the environment affected lifespan through various mechanisms [27]. Additionally, our study underscored the substantial detrimental impact of AMP stress on the reproductive capacity of *Drosophila*, aligning with prior research indicating decreased mating duration and fertility in *Drosophila* under Pb (lead) stress [17]. It may have been due to increased latency and shorter mating duration with AMP-treated samples [28], which suggests that AMP disrupted estrogen synthesis, leading to ovarian necrosis [23]. Furthermore, our study observed decreased viability of ovarian and testicular cells in F_4_ generation AMP-exposed samples, paralleling the cytotoxic effects of ampicillin on human cells reported by Smith et al. [29].

Interestingly, AMP exposure was found to increase mouth hook movement in larvae, possibly due to induced feeding at AMP stress as compensational recovery from damage created by stressor. Extensive exploration of the toxicological effects of ampicillin across various organisms has been documented [30]. For instance, Zhank et al. [30] elucidated significant reductions in eclosion rates and alterations in feeding behaviour in *Drosophila melanogaster* following ampicillin exposure, which is similar to the present finding. Antibiotics can induce oxidative stress and caused lipid damage [31] in *Drosophila melanogaster* leading to the decreased in the fat body. Additionally, antibiotics causes a series of oxidative damage to DNA and protein [31]. Ciprofloxacin exposure in *Drosophila* delay developmental progression like twice the puparium formation and adult stage compared to control [32]. This abnormal behaviour might be due to repulsion from antibiotic as an environmental cue [33].

For understanding the above changes implicated by ampicillin stress, which potentially influenced gene expression patterns without modifying the DNA sequence. DNA methylation stood as a pivotal epigenetic modification, orchestrating specific gene silencing, triggering apoptosis, and restraining mitosis and transcription to combat toxicity stress like Cd, Pb, etc [17]. Our study found that female *Drosophila* exposed to AMP exhibited downregulation of *dMBD2/3* and *dDnmt2* expression. This downregulation was transmitted to the subsequent generation of rescue flies. However, by the next two generation, methylation levels had reverted to normal which was similar to the results obtained by Yang et al. [34] that even after removal of cadmium stress, the effect of cadmium on fertility, lifespan, can still be transmitted to the offspring from two generations and one generation respectively [34]. The induction of alterations in DNA methylation patterns and histone modifications by AMP in human cells were reported in previous study [24]. This suggested a disruptive potential of ampicillin on epigenetic mechanisms, affecting cellular function and health outcomes.

Antibiotic exposure has been shown to induce oxidative stress, leading to lipid damage in *Drosophila melanogaster*, which in turn results in a reduction of the fat body [31]. Additionally, antibiotics can cause oxidative damage to DNA and proteins [31] and may also alter the relative expression of genes [35]. A decrease in DNA methylation can lead to the upregulation of apoptosis-related genes such as p53, which is associated with a shortened lifespan in *Drosophila* [36]. Moreover, environmental pollutants such as heavy metals and antibiotics can induce epigenetic modifications in CpG islands within promoter regions, potentially affecting apoptosis-related genes. These epigenetic modifications induced by environmental factors may be heritable [37]. In *Folsomia candida* and *Caenorhabditis elegans*, exposure to environmental pollutants, including insecticides and heavy metal stress, has been reported to induce epigenetic changes [38]. The decrease in DNA methylation levels may have prompted heightened expression of apoptosis-related genes such as *p53*, consequently leading to a shortened lifespan in *Drosophila*. Recent studies have delved into the interplay between DNA methylation, apoptosis, and ampicillin, revealing potential mechanisms underlying the antibiotic's cytotoxic effects. For example, explored ampicillin's impact on DNA methylation patterns in human cells, highlighting alterations linked to apoptotic pathways, indicative of a connection between ampicillin-induced changes in DNA methylation and apoptotic activation [39]. Our study unveiled a significant increase in apoptotic cell death in treated females, while no significance was observed in males.

AMP stress induced competition among bacterial communities triggered by microbiota dysbiosis and ampicillin exposure in *Drosophila melanogaster*. This resulted in a reduced bacterial population in the treated group, with *Wolbachia* sp. emerging as the dominant species, suggesting a potential link between antibiotic exposure and microbial shifts, as also reported by Singh et al. [21]. The presence of *Wolbachia* in treated samples appeared to have multiple effects on the host population, including potential negative impacts on sperm function and male fertility, induction of oxidative stress, and alterations in dopamine production, which may influence juvenile hormone levels in female flies [38]. The dominance of a single gut microbe can ultimately lead to apoptosis, increased host mortality, and reduced overall health and fitness, as seen in *Gluconobacter* sp. strain EW707 [40]. The *Drosophila* gut plays a crucial role in producing microbial reactive oxygen species (ROS) and antimicrobial peptides (AMPs), which contribute to the activation of the host’s antimicrobial defense [41]. These findings highlight that shifts in the intestinal microbiota reflect the ongoing competition among bacterial communities induced by microbiota dysbiosis. Our study further revealed that AMP exposure downregulated antimicrobial peptide expression in males, whereas it was upregulated in females. This upregulation in females may be associated with an increase in specific gut microbes, such as *Wolbachia*, which could trigger antimicrobial peptide overexpression as a compensatory response to stress, thereby enhancing tolerance. Singh et al. [21] similarly reported a significant decrease in antimicrobial peptide expression in ampicillin-treated males, while females exhibited upregulation with the increased in Wolbachia sp. in the Ampicillin exposed flies.

In the broader context, there existed at least two plausible mechanisms facilitating such non-Mendelian inheritance of phenotypic traits: firstly, alterations in the parental metabolic system may have triggered foetal developmental exposure in the subsequent generation; and secondly, epigenetic inheritance played a significant role [32]. Our findings demonstrated alterations in the expression of methylation-related genes (*dMBD2/3* and *dDnmt2*) in AMP-treated flies and their progeny. Thus, we postulated that the observed lifespan reduction, fertility decline, and multigenerational transmission in *Drosophila* were likely attributed to epigenetic modifications. Nevertheless, numerous unanswered queries persisted regarding the transgenerational inheritance of epigenetic variation, epigenetic memory etc. These included inquiries into how acquired epigenetic markers evaded reversal, whether diverse environmental factors exhibited species-specific effects, and the degree of stability in transgenerational inheritance. Addressing these issues necessitated further investigation.

## 5 Conclusion

Our findings underscore the multigenerational impact of AMP exposure on the lifespan, fertility, and gene expression profiles related to apoptosis and methylation in *Drosophila*. We observed significant reductions in lifespan and fertility in *Drosophila* subjected to AMP stress over five consecutive generations (F_0_ to F_4_) across treatment groups. Additionally, mRNA levels of apoptosis-related genes, namely *p53* were increased in female flies, whereas methylation-related genes *dDnmt2* and *dMBD2/3*, were downregulated following AMP exposure. Moreover, these gene expression alterations persisted in the offspring for up to three generations after the cessation of AMP exposure, suggesting that AMP exerts regulatory effects on gene expression via epigenetic mechanisms. In light of these findings, it is imperative to conduct long-term multigenerational experiments to comprehensively assess the enduring effects of environmental pollutants on organisms. Such endeavours are crucial for devising effective strategies to prevent and mitigate environment pollution.

In summary, our study reveals that the AMP stress increased the antimicrobial peptide production to tolerate the stress and AMP downregulates the *dDnmt2* which may result in the shortening of the telomere and leads to increase in the apoptosis which leads to shorten the life span of the *Drosophila melanogaster*. This suggests that antibiotic pollution could have long-term effects on the reproductive system and immune systems of an organism, highlighting the potential risk of antibiotic bioaccumulation in higher organisms like humans due to persistent environmental pollution by the healthcare and agriculture industries. Moreover, this study encourages further research on antibiotic-induced epigenetic changes and their inheritance and studying the broader impact of antibiotic resistance on other model organisms and on human health.

## Declarations

none

## Ethics approval

Not applicable

## Funding declaration

The authors express their gratitude to the Indian Institute of Science (IISc) for offering essential facilities to conduct this research. The first author acknowledges the financial support received through the 10.13039/100012359DBT-RA post-doctoral program in Biotechnology and Life Science, including fellowship and contingency, under Grant ID-DBT-RA/2022/January NE/994.

## Consent to participate

Not applicable

## Consent for publication

All authors have provided their consent for the publication of this manuscript.

## CRediT authorship contribution statement

**Nongthomba Upendra:** Writing – review & editing, Supervision, Methodology, Formal analysis, Conceptualization. **Devi Manoharmayum Shaya:** Writing – review & editing, Methodology, Conceptualization. **Jain Sakshi:** Writing – original draft, Data curation. **Pathak Dhruv:** Data curation. **Singh Asem Sanjit:** Writing – review & editing, Writing – original draft, Methodology, Investigation, Funding acquisition, Formal analysis, Data curation, Conceptualization.

## Declaration of Competing Interest

The authors declare that they have no known competing financial interests or personal relationships that could have appeared to influence the work reported in this paper

## Data Availability

Data will be made available on request.
